# Treatment of deep-seated facial microcystic lymphatic malformations with intralesional injection of pingyangmycin

**DOI:** 10.1097/MD.0000000000004790

**Published:** 2016-09-16

**Authors:** Hai Wei Wu, Xuan Wang, Jia Wei Zheng, Hai Guang Zhao, Jing Ge, Ling Zhang, Yan An Wang, Li Xin Su, Xin Dong Fan

**Affiliations:** a Department of Oral and Maxillofacial Surgery, Shanghai Ninth People's Hospital, College of Stomatology, Shanghai Jiao Tong University School of Medicine, Shanghai, China; b Department of General Dentistry, Shanghai Ninth People's Hospital, College of Stomatology, Shanghai Jiao Tong University School of Medicine, Shanghai, China; c Department of Vascular Surgery, Shanghai Ninth People's Hospital, Shanghai Jiao Tong University School of Medicine, Shanghai,China; d Department of Interventional Radiotherapy, Shanghai Ninth People's Hospital, Shanghai Jiao Tong University School of Medicine, Shanghai,China.

**Keywords:** intralesional injection, lymphatic malformations, microcystic, pingyangmycin

## Abstract

Treatment of microcystic lymphatic malformations (LMs) is still a great challenge to physicians in the field of managing vascular anomalies. Several kinds of treatment have been proposed for microcystic LMs, but the responses to these treatment modalities vary considerably among individuals. The aim of the study was to investigate the safety and efficacy of intralesional injection of pingyangmycin for microcystic LMs located in the deep facial region.

Twenty-one consecutive patients with deep-seated facial microcystic LMs were treated with intralesional injection of pingyangmycin between March 2010 and April 2015. The patients received 2 to 8 injections, and the average session was 3.7. The therapeutic efficacy was accessed on the basis of the imaging findings and clinical measurements.

Among the 21 patients, the clinical responses were excellent in 7 patients (33.33%), good in 9 patients (42.86%), fair in 3 patients (14.29%), and poor in 2 patients (9.52%). No severe side effects were encountered. Furthermore, therapeutic outcomes were significantly associated with lesion location (*P* = 0.006) and number of injections (*P* = 0.003).

Our study supports that sclerotherapy with pingyangmycin is safe and effective for the treatment of deep-seated facial microcystic LMs.

## 1 Introduction

Lymphatic malformations (LMs) are one common type of congenital vascular lesions, occurring in 1 of 20,000 in children admitted to hospital.^[1]^ The cause of LM has not been well elucidated, but LMs are commonly thought to arise from the failure of embryonic lymphatic tissue to communicate with the venous system.^[2]^ These lesions are characterized by abnormal channels and cysts fulfilled with lymphatic fluid. LM is most commonly seen in the lymphatic-rich head and neck. The description of the size and locations of these cystic lymphatic lesions allows classifications of LMs into 3 types: macrocystic, microcystic, and mixture of macrocystic and microcystic components. Macrocystic lesions have cysts with volume ≧ 2 cm^2^, while all cysts of microcystic lesions have volume <2 cm^2^. Lesion type is of vital importance in predicting the therapeutic effect of LMs and there is a general consensus that treatment of macrocystic LMs could achieve greater success rates than that of microcystic LMs.^[3,4]^ Clinically, ultrasonography and magnetic resonance imaging (MRI) are widely applied in the diagnosis and evaluation of LM, which favors in delineating LM extension and relationship to surrounding structures.^[5]^ As for histopathological diagnosis, podoplanin (D2–40) and lymphatic vessel endothelial hyaluronan receptor (LYVE)-1 are the immunohistochemical markers on histology to identify the lymphatic endothelial cell.^[6]^ Clinical symptoms of LMs depend on the size, location, extension, and relationships with surrounding structures of the lesions. Functional disturbances and cosmetic disfigurements indicate for the need of treatment, including airway obstruction, severe swelling, repeated infections, bleeding, and compression of vital structure.^[1]^

Currently, there is no gold standardization of treatment pattern for LMs. In the published literature, several kinds of treatment have been proposed for LMs, including sclerotherapy, radiofrequency ablation, carbon dioxide laser, medical treatment, and surgery. Recently, sclerotherapy gains more popularity in treating microcystic LMs and proves to be rather effective.^[7–9]^ Sclerotherapy, as a less invasive intervention than surgery, has gained more popularity in the treatment of LMs though intralesional injection of sclerosants, such as ethanol, OK-432, doxycycline, bleomycin, and pingyangmycin.^[10–15]^ Ethanol is commonly used as a sclerosant for LMs, but its application in children is limited because of its neurolytic and systemic effects.^[16]^According to an analysis of the outcomes of sclerotherapy of LMs, most permanent complications occurred in the use of ethanol than other sclerosants.^[17]^ Another sclerosant OK-432, derived from killed bacterium *Streptococcus pyogenes*, was used for treatment of LMs in many counties. It could activate the immune responses and lead to the inflammation of lymphatic endothelial cells.^[18]^ One major complication of OK-432 use is delayed swelling and higher fever. Ghaffarpour et al^[11]^ reported 5 patients with readmisson to the hospital as a result of major swelling after injection. In addition, the use of OK-432 is forbidden in patients who are allergic to penicillin. Doxycycline is a kind of broad-spectrum antibiotics, which could inhibit lymphatic endothelial cell proliferation and downregulate lymphangiogenesis. Percutaneous injection of doxycycline is effective and safe for patients with LMs but is not suitable for outpatient treatment because injection of doxycycline is usually performed under general anesthesia.^[19]^ Pingyangmycin is bleomycin A5 isolated from bleomycin produced by *Streptomyces pingyangensisn*. Pingyangmycin and bleomycin are chemotherapeutic drugs that share similar chemical components but not the terminal amine moiety.^[20]^ Pingyangmycin has been widely applied in the treatment of vascular malformations in our department because of its safety and low cost.

It should be addressed that responses to different treatment modalities vary considerably among individuals. In particular, microcystic LMs have multiple internal separations and usually infiltrate into the deep tissue, which makes it more difficult and challenging to treat. All above sclerosants have shown obvious efficacy for macrocystic LMs but an uncertain effect for microcystic LMs. However, most of effective cases published in the previous studies are superfacial microcystic LMs that are located in the surface of oral mucosa. Whether sclerotherapy could be an optimal selection for the treatment of deep-seated microcystic LMs requires further investigations. In the present study, we evaluated the effectiveness of intralesional injection of pingyangmycin for the treatment of deep-seated facial microcystic LMs.

## 2 Materials and methods

### 2.1 Patients

Between March 2010 and April 2015, 21 patients with microcystic LMs located in the deep facial region were recruited in Department of Oral and Maxillofacial Surgery, Shanghai Ninth People's Hospital, College of Stomatology, Shanghai Jiao Tong University School of Medicine. Approval for the treatment was obtained from the Institute Review Board of our hospital and informed consents were signed by the patients or the parents of the patients. The study was performed in accordance with approved guidelines and regulations. The diagnosis of deep-seated facial microcystic LMs was confirmed by patient history, physical examination, Doppler ultrasonography scan, and MRI in selected cases. To assess the efficacy of the primary treatment with sclerotherapy, the patients who previously underwent other treatments were excluded from the study.

### 2.2 Preparation before injection

For treatment safety, blood test and electrocardiogram were performed to exclude systemic disease before the injection. All procedures were performed without general anesthesia at the outpatient clinic. Before sclerotherapy, 8 mg pingyangmycin (acquired from Tianjin Taihe Pharmaceutics Co., Ltd., Tianjin, China) was dissolved in 4 mL saline, 3 mL 2% lidocaine, and 1 mL dexamethasone (5 mg). The final concentration was 1 mg/mL.^[20]^

### 2.3 Dosage

The dosage of pingyangmycin was determined by the size of the lesion and the amount of the lesion area of 1 cm^2^ ranged from 1 to 2 mg. The maximum dosage of single treatment could not exceed 8 mg in adults and 4 mg in children. Considering that deeper microcystic LMs were usually extensive and diffuse, sclerotherapy was performed in multiple sessions. The total dosage should be less than 40 mg in adults and 20 mg in children over 1 year.

### 2.4 Pingyangmycin injection

The injection of pingyangmycin (1 mg/mL) was performed through 21-gauge needle. Before injection, the depth of needle puncture in different areas was deduced according to the imaging measurement. The lesion areas were sterilized strictly, as the lesions in this study were all located deeply. The needle was directly introduced through the surface and advanced into the directed depth in the deep tissue plane. As there were multiple intervals and cysts in the microcystic LMs, pingyangmycin was injected at multiple sites so that more pingyangmycin could infiltrate into the lesions. After injection, the injection sites were compressed locally for 5 to 10 minutes. Repeated injections were performed 3 to 4 weeks later according to the individual performance and response.

### 2.5 Outcome measurement

The follow-up period ranged from 6 months to 4 years, with a mean follow-up time of 33 months. Postoperative follow-up data for each patient included the frequency of treatment, the efficacy of treatment, and complications. The therapeutic efficacy was accessed by other 3 independent physicians from the Department of Oral and Maxillofacial Surgery. The response rate was evaluated by the imaging findings and clinical measurements. The response rate was graded as follows: excellent response (apparently cured and resolution in lesion size of >90%), good response (marked improvement in appearance and resolution in lesion size of 50–90%), and fair responses (minor improvement in appearance and resolution in lesion size of <50%) and poor response (no reduction or even enlargement in size).^[7,20,21]^

### 2.6 Statistical methods

Statistical calculations were performed using the SPSS software package (version 16.0; SPSS, Chicago, IL). *P* values were calculated by comparing different groups with diverse responses (excellent + good response vs fair response vs poor response). *P* values <0.05 were considered as significant. All statistical tests were 2-tailed, and ɑ was set at 0.05. Data were calculated according to a nonparametric 1-way analysis of variance. The Mann–Whitney *U* test was used to compare differences between groups.

## 3 Results

### 3.1 Clinical characteristics of patients

The clinical characteristics of patients are listed in Table 1. Of all patients, 14 were males and 7 were females. Age ranged from 6 months to 27 years with a mean age of 75 months and a median age of 36 months. The lesions were located at local or diffuse sites: 6 in the right cheek, 6 in the left cheek, 5 in the upper lip, 2 were diffuse involving cheek and lip, and 2 were diffuse involving cheek, lip, and infraorbital region. The size of the lesions was 1 cm × 1.5 cm to 10.7 cm × 11.3 cm. Patients received 2 to 8 injections, and the average session was 3.7.

**Table 1:**
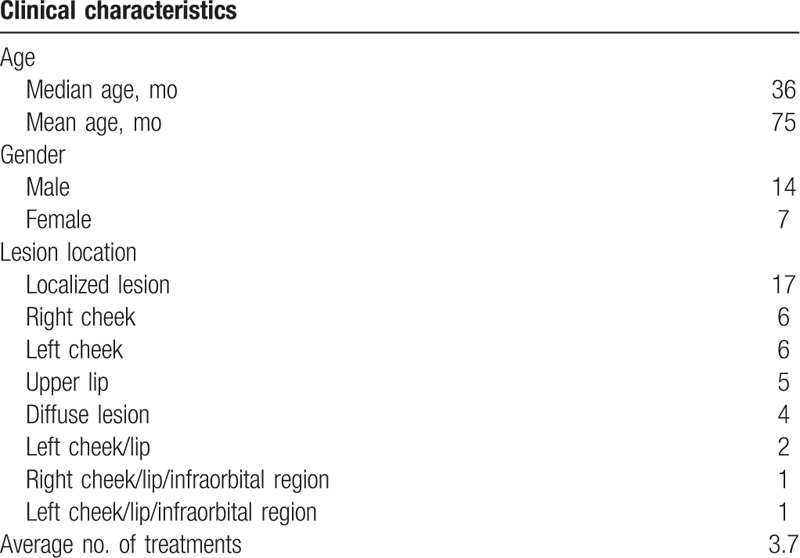
The clinical characteristics of patients.

### 3.2 Therapeutic outcomes

**Figure 1:**
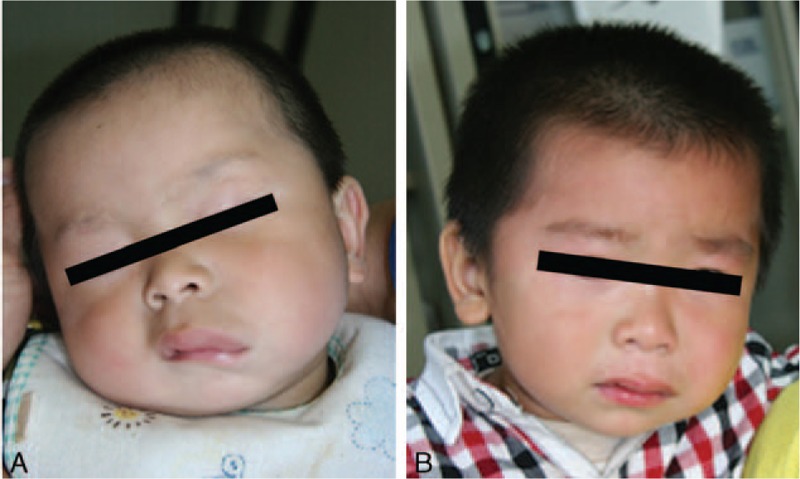
Clinical photographs of a male patient with deep microcysitic LMs located in the left cheek. (A) 7 months of age; before starting sclerotherapy with pingyangmycin; (B) 28 months of age; after 4 injections of pingyangmycin, the lesion was significantly reduced in size and facial appearance was improved.

**Figure 2:**
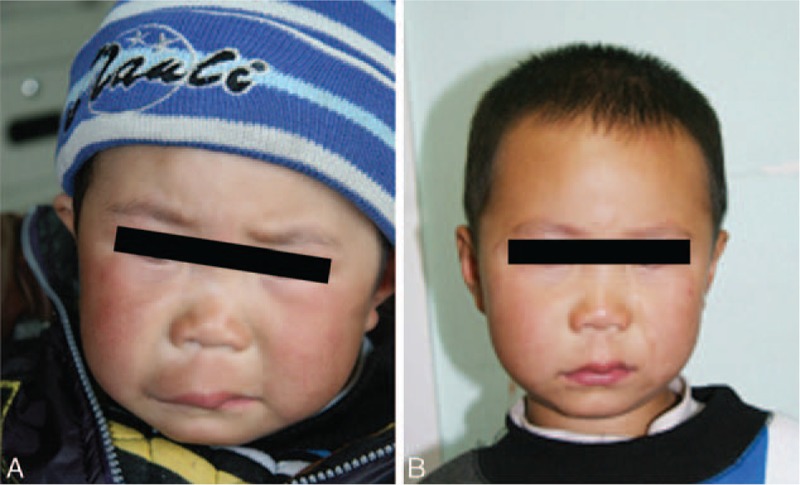
Clinical photographs of a male patient with deep microcysitic lymphatic malformations located in the upper lip. (A) 12 months of age; before starting sclerotherapy with pingyangmycin; (B) 31 months of age; after 3 injections of pingyangmycin, symmetric appearance of the upper lip was obtained.

**Figure 3:**
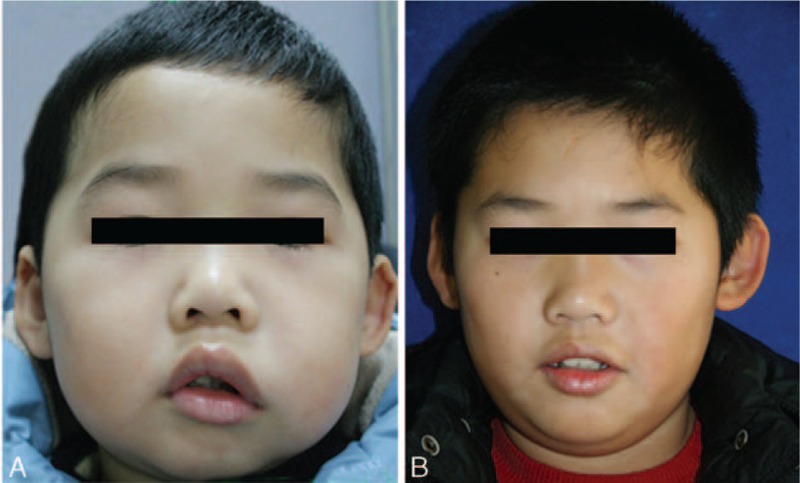
Clinical photographs of a male patient with deep microcysitic lymphatic malformations located in the diffuse area involving lip and cheek. (A) 3 years of age; before starting sclerotherapy with pingyangmycin; (B) 6 years of age; after 6 injections of pingyangmycin, the diffuse lesion resolved completely with normal appearance of the left lip and cheek.

**Figure 4:**
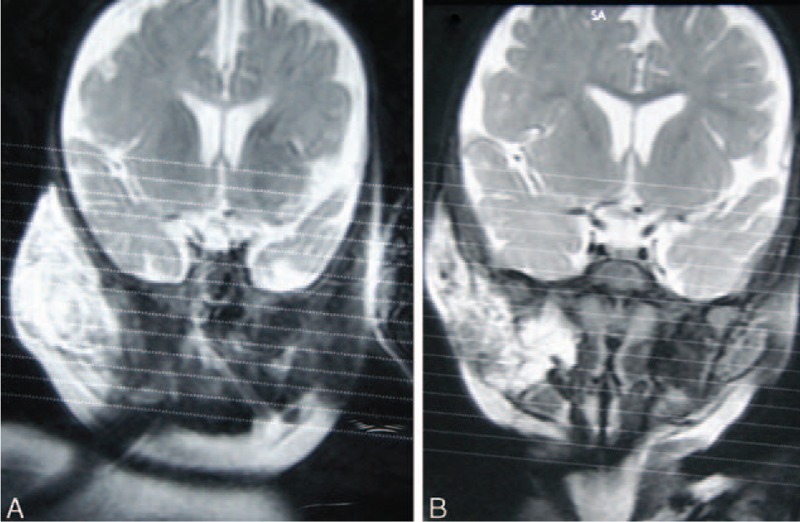
T2-weighted magnetic resonance imaging of a male patient with deep microcysitic lymphatic malformations located in the right cheek. (A) 6 months of age; before starting sclerotherapy with pingyangmycin; (B) 12 months of age; after 3 injections of pingyangmycin, obvious decrease in size and signal of the lesion was shown in T2-weighted magnetic resonance imaging.

Table 2 outlines the therapeutic responses of the lesions located at different sites. Overall, the clinical responses were excellent in 7 patients (33.33%) (excellent responses in typical cases is shown in Figs. 1–3), good in 9 patients (42.86%) (good responses in typical cases is shown in Fig. 4), fair in 3 patients (14.29%), and poor in 2 patients (9.52%). Among 17 patients with local lesions, 15 patients (88.24%) achieved excellent or good responses and 2 patients achieved fair responses. The therapeutic effect of lesions located in the diffuse area was poor, 3 in 4 patients had fair or poor responses, and they are now taking sirolimus orally to improve their facial appearance.

**Table 2:**
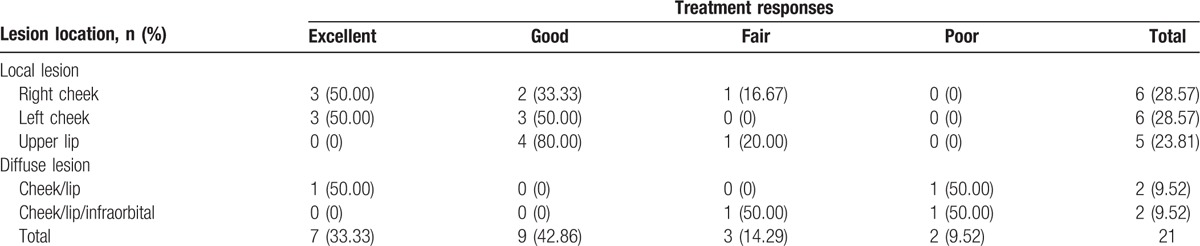
The therapeutic outcomes of patients.

### 3.3 Complications

All patients experienced slight swelling after injection, which was relieved in 7 to 10 days. Two patients had mild fever around 38°C on the day following injection and no further treatment was proposed. No patients experienced other side effects due to pingyangmycin treatment, such as gastrointestinal reaction, nerve damage, pulmonary fibrosis, and acute allergic reactions.

### 3.4 The impact of clinical variables on therapeutic outcome

As summarized in Table 3, 7 variables were tested in this study, including gender, age at first injection, duration of the follow-up, lesion location, lesion side, lesion volume, and number of injections. The therapeutic outcomes were significantly associated with lesion location (*P* = 0.006) and number of injections (*P* = 0.003), but not gender (*P* = 0.617), age at first injection (*P* = 0.308), duration of the follow-up (*P* = 0.725), lesion side (*P* = 0.929), and lesion volume (*P* = 0.317).

**Table 3:**
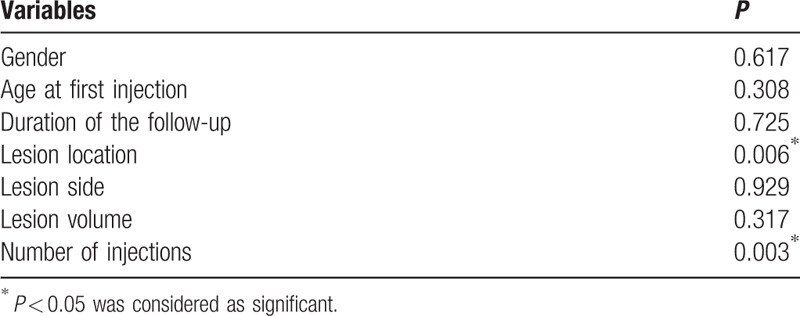
Analysis of clinical variables for predicting therapeutic outcome.

## 4 Discussion

LM is a benign lesion arising from abnormal development of lymphatic system. The lesions could be seen in any lymphatic-rich area, such as neck, axilla, groin, mediastinum, and retroperitoneum, but most commonly found in head and neck.^[22]^ These lesions could involve different tissue planes, including mucosa, skin, subcutaneous tissue, and muscle. Although the molecular mechanism of LM is unknown, the emerging of more ideal laboratory models of LM and substantial studies on the genetics of lymphatic anomalies gradually unveils the secrets of LM.^[23]^ According to an assessment of disease impacts reported by patients and parents, different stages of head and neck LMs (according to the LM staging system proposed by de Serres et al^[24]^) could affect several domains of daily life, including pain, swelling, infection, prolonged or frequent sickness, social stigma, breastfeeding, etc.^[25]^ Several therapeutic interventions have been proposed in the treatment of LMs, including aspiration, raidofrequency ablation, laser, and sclerotherapy.^[1]^ However, there is no consensus as to which is the optimal treatment approach. The selection of treatment modality depends on the type, size and location of the lesion, patients’ physical status, and the severity of complications.

Surgical resection has been a traditional choice of treatment of LM over the past decades and achieved fair results for local macrocystic LMs. However, deep and extensive microcystic LMs are prone to infiltration of deep tissue planes and envelopment of vital structures, which makes complete surgical resection more difficult or even impossible due to anatomic restriction. Incomplete resection is associated with higher recurrence rates and complications.^[26]^ Furthermore, nerve function might be permanently damaged during surgical resection.^[27]^ Taking into account that LM is a benign lesion, permanent damage to normal organs could not be accepted by most patients. Even though surgical technique has achieved great progress, postoperative complications could not be avoided completely, including pain, infection, scarring, and disfigurement. Among the patients involved in this study, primary surgical resection of the lesions was not recommended, as it has a potential risk of facial disfigurement and facial nerve palsy. However, it should be pointed out that surgery is still a reserved means after the failure of other treatments or a supplement with other therapies.

There is a consensus that microcystic LMs tend to be more resistant to treatment possibly because there are no obvious cysts to target within the lesions. Balakrishnan et al^[3]^ reported that patients with microcystic LMs were more likely than patients with macrocystic LMs to need further treatment after the first intervention. Consequently, the treatment of microcystic LMs stills remains a great challenge to physicians in the field of managing vascular anomalies.

Recently, sclerotherapy with bleomycin or pingyangmycin is becoming more popular in attempting to treat microcystic LM in several countries.^[28]^ Bleomycin was initially utilized as a chemotherapeutic agent and Yura et al^[14]^ firstly reported its application in the treatment of LMs. Bleomycin and pingyangmycin have similar chemical structures and both of them are inexpensive, safe, and easy to manipulate. We have used pingyangmycin as the sclerosant for LMs since 1996 at outpatient clinic and treated over 300 patients with vascular anomalies. Bleomycin could inhibit DNA synthesis, destroy the endothelial junction, and promote endothelial cells transforming into fibroblasts.^[29]^ The histologic change of resected tissues after sclerotherapy with pingyangmycin showed that lymphatic endothelial cells were destroyed and lymphatic vessels were obstructed.^[7]^

Our previous study found that pingyangmycin is effective in treating tongue microcystic LMs.^[9]^ Chaudry et al^[8]^ reported treatment of microcystic or mixed LMs with bleomycin and 31 patients underwent percutaneous image guided injection of bleomycin. Of them, 12 (38%) showed complete reduction in size; 18 (58%) showed partial reduction in size; only 1 showed no responses.^[8]^ Bai et al^[7]^ documented that 78% of patients with microcystic LMs achieved obviously therapeutic effect after injection of pingyangmycin. In the study by Yang et al,^[30]^ they successfully treated periorbital microcystic LMs through combination of surgery and injection of bleomycin. In our present study, 33.33% of 21 patients achieved excellent outcome and 42.86% of 21 patients achieved good outcome. All these studies demonstrated that bleomycin and pingyangmycin proved to be a potentially effective agent in treating microcystic LMs.

In this series, we evaluated the effectiveness of pingyangmycin sclerotherapy for deep facial microcystic LMs. Superficial microcystic LMs in the face and oral cavity usually manifest as tiny vesicles, while deep facial microcystic LMs can lead to severe facial deformity and bulking of involved tissues indicating need for treatment. Compared with superficial microcystic LMs, deep-seated microcystic LMs are more difficult to treat, as deep lesions tend to infiltrate into subcutaneous tissue and it is hard to deliver the drug to the proper area. To improve the treatment outcomes, we assessed the depth and size of the lesions according to ultrasonography scan and tried to disseminate the solution into the assigned region. Although there is no dominant cyst in the lesions, pingyangmycin could permeate the interstitial stroma and inhibit lymphangiogenesis. Recently, image-guided technique under general anesthesia or intravenous sedation is popular in treating deep lesions.^[8]^ Considering that many parents of the children are concerned over the safety and potential complications of general anesthesia, our method is more convenient to practice in clinic.

**Figure 5:**
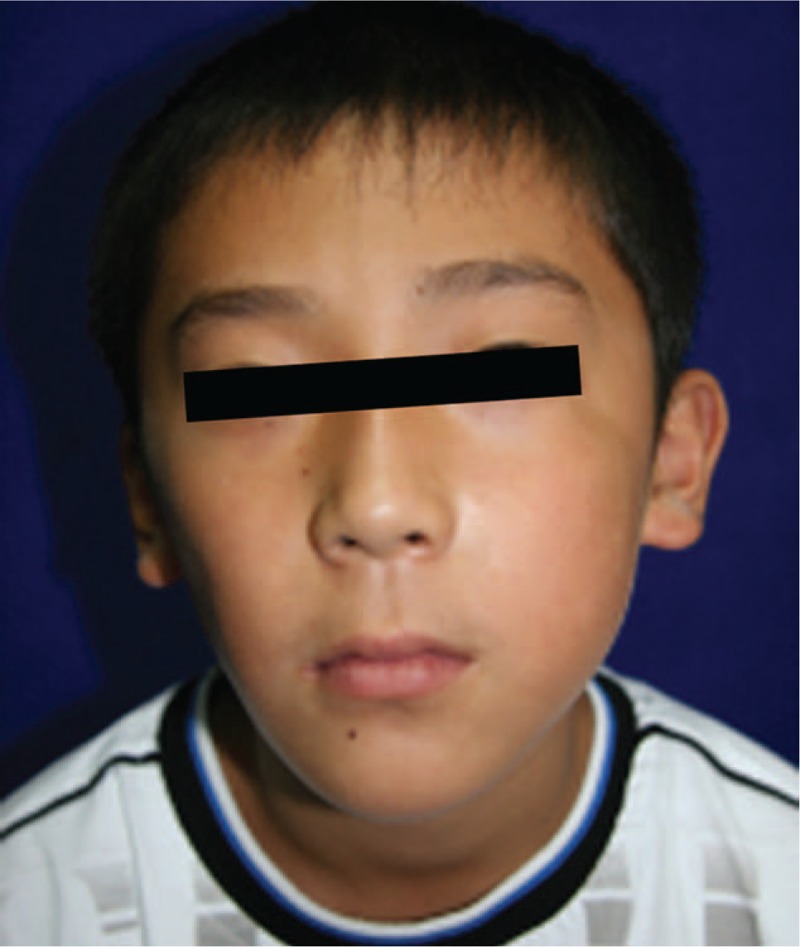
A patient with obvious tissue atrophy in the right cheek after 10 injections of pingyangmycin at other clinic. Facial appearance looks obviously asymmetric.

In our study, diffuse lesion and more injections of pingyangmycin predicted a less favorable outcome, which was consistent with the results of long-term study of sclerotherapy with OK-432 for treating LM.^[11]^ Three in 4 patients with diffuse lesions involving nearly the entire face had fair or poor therapeutic effects. They were given sirolimus at a dose of 0.8 mg/m^2^, taken twice daily at the internal of 12 hours.^[31]^ The final results are waiting to be estimated. If the improvement was not distinct, surgical remodeling or debulking will be conducted when the child is over 3 years old. Furthermore, number of injections was associated with therapeutic outcomes. Patients obtaining fair or poor outcome required more repeated injections (mean, 6.4 sessions) than patients acquiring excellent or good outcome (mean, 2.8 sessions). In addition, the average injection (mean, 3.7 sessions) in this study was higher than that in our previous study (mean, 3.0 sessions),^[9]^ suggesting that deep microcystic LMs are much harder to treat than superficial microcystic LMs. It should be addressed that transient facial symmetry with too many injections should not be attempted, because repeated injection is inevitable as the child grows up, and too many injections of pingyangmycin in a short time can cause soft tissue atrophy and cosmetic problems (1 typical case is shown in Fig. 5). As summarized in Table 3, gender, age at first injection, duration of the follow-up, lesion side, and lesion volume were not factors influencing therapeutic outcome. However, our study had limitations of patient number and a long-term study based on a large samples data is mandatory in the future.

After sclerotherapy, mild or moderate swelling was the main complication, which could be resolved in 7 to 10 days. Considering that the lesions in this series were located in the deep facial tissue, care should be taken not to damage the nerve. Fortunately as reported by Karavelioglu et al,^[32]^ subcutaneous injection of pingyangmycin did not cause facial nerve palsy. No other severe complications, such as pulmonary fibrosis, gastrointestinal reaction, and acute allergic reactions, occurred after treatment due to the control of concentration and dosage.

In conclusion, the present study summarized our experience of using pingyangmycin sclerotherapy to treat deep-seated facial microcystic LMs. Although our sample number is small, the acceptable results support that sclerotherapy with pingyangmycin is effective for the treatment of microcystic LMs located in the deep facial region.
